# Current progress and controversies in thoracic ossification of the ligamentum flavum: pathogenesis, diagnosis and surgical management

**DOI:** 10.3389/fsurg.2026.1896885

**Published:** 2026-10-01

**Authors:** Guoxing Ding, Huijing Shang, Fuhai Yan, Weijie Cui, Zhi Li, Yiheng Zhang, Zhan Wang, Jie Wen, Yifan Feng, Junxiao Su, Hui Zhang

**Affiliations:** 1 Department of Pain Medicine, Liangzhou Hospital, Wuwei, Gansu, China; 2 The First Clinical Medical College of Gansu University of Traditional Chinese Medicine, Lanzhou, Gansu, China; 3 Department of Spine Surgery, Gansu Provincial People’s Hospital, Lanzhou, Gansu, China; 4 College of Basic Medicine, Mudanjiang Medical University, Mudanjiang, Heilongjiang, China; 5 The First Affiliated Hospital of Tsinghua University, Beijing, China

**Keywords:** research progress, thoracic disc herniation, thoracic ossification of ligamentum flavum, thoracic ossification of posterior longitudinal ligament, thoracic spinal stenosis

## Abstract

Thoracic ossification of ligamentum flavum (TOLF) is the primary pathological driver of thoracic spinal stenosis (TSS), characterized by insidious clinical onset, high surgical complexity, and a elevated risk of permanent neurological deficits. This systematic review synthesizes the epidemiological landscape and genetic, mechanical, and inflammatory pathogeneses ofTOLF. We provide a comprehensive evaluation of current radiological classification systems and critically appraise different decompressive procedures—ranging from classic open laminectomies to emerging minimally invasive biportal and uniportal endoscopic techniques. By assessing surgical outcomes, complications (e.g., cerebrospinal fluid leakage), and clinical indicators, this review aims to provide clinicians with an evidence-based framework for individualized surgical decision-making in managing TOLF.

## 1 Literature search strategy

A systematic literature search was strictly performed in accordance with the Preferred Reporting Items for Systematic Reviews and Meta-Analyses (PRISMA) guidelines to ensure the transparency and reproducibility of the present study. Public databases including PubMed, Embase, Web of Science, and the Cochrane Library were comprehensively searched for relevant literature published from January 2000 to December 2025. The major search terms were as follows: “thoracic spinal stenosis”, “thoracic ossification of the ligamentum flavum (TOLF)”, “thoracic ossification of the posterior longitudinal ligament (TOPLL)”, and “thoracic disc herniation”, supplemented by keywords related to pathogenesis, imaging classification, surgical intervention, postoperative JOA score improvement, and surgical complications.

Inclusion criteria:

(1) Clinical studies, translational basic researches, systematic reviews, and meta-analyses focusing on TOLF-induced thoracic spinal stenosis; (2) Studies that clearly reported surgical procedures, clinical efficacy, perioperative complications, and long-term follow-up outcomes of TOLF treatment; (3) Studies with complete quantitative data on JOA improvement rate, complication rate, or reoperation rate; (4) Peer-reviewed articles with full texts available in English or Chinese.

Exclusion criteria:

(1) Case reports with a sample size of fewer than 10 patients, letters, and conference abstracts without complete full-text data; (2) Studies merely focusing on isolated TOPLL, thoracic disc herniation, or mixed-type thoracic spinal stenosis without independent subgroup analysis of TOLF; (3) Duplicate publications and low-quality studies characterized by incomplete core data and flawed research design; (4) Pure animal experimental studies lacking clinical translational value for TOLF treatment.

TSS refers to a series of neurological dysfunction syndromes caused by hyperplasia, hypertrophy, ossification or calcification of osseous or soft tissue structures within the thoracic spinal canal, which compress the thoracic spinal cord and nerve roots. Compared with cervical and lumbar spinal stenosis, TSS is relatively rare, accompanied by high surgical risk, high disability rate and relatively poor prognosis (1, 2). The thoracic spinal canal possesses unique anatomical structures. Firstly, the thoracic spinal cord has weak blood supply, making it vulnerable to ischemic injury. Secondly, the physiological kyphosis ofthe thoracic spine limits the backward displacement of the spinal cord when the ventral side of the thoracic spinal canal is compressed (3–5). In addition, TSS is a slowly progressive degenerative disease that may remain asymptomatic for a long time, resulting in irreversible neurological damage caused by delayed diagnosis (6). Moreover, the thoracic spinal cord has extremely low tolerance to mechanical compression, and any minor operational errors during surgical decompression may lead to catastrophic neurological impairment. In recent years, with the aggravation of global population aging and the popularization of advanced imaging diagnostic techniques such as MRI and CT, the clinical detection rate of TSS has been increasing year by year (7). Its main pathological mechanisms include thoracic ossification of the ligamentum flavum (TOLF), thoracic ossification of the posterior longitudinal ligament (TOPLL), thoracic disc herniation (TDH) and hyperplasia and aggregation of facet joints, among which TOLF is the most common type. Previous studies (8–10) have reported that the prevalence ofTSS ranges from 20% to 60%, which is more common in East Asia. However, it is clinically crucial to distinguish between asymptomatic radiological findings and symptomatic clinical disease. Conservative treatment achieves poor efficacy for TSS, and surgical intervention is usually required to relieve spinal cord compression and preserve neurological function. Traditional posterior laminectomy is a classic surgical method, yet open surgery has disadvantages such as severe tissue trauma, long hospital stay and high infection risk. Besides, lesions are often closely adhered or even fused with the dura mater, leading to a persistently high incidence of surgical complications including cerebrospinal fluid leakage (CSFL) and postoperative neurological deterioration (PND) (10). In recent years, spinal surgery has developed towards minimal invasiveness and precision. The wide application of full-spine endoscopy, intraoperative navigation, robot-assisted surgery, ultrasonic bone scalpel and intraoperative neurophysiological monitoring (IONM) has greatly enriched the treatment strategies for TSS and significantly improved the safety and efficacy of surgery. Based on the latest clinical research evidence, this paper reviews the epidemiology, pathological mechanisms, classification and treatment of TSS in detail, so as to provide decision-making references for clinicians in the treatment of TSS.

## 2 Epidemiology

TSS is mainly caused by TOLF, which accounts for 61% of all TSS cases (11). It has a higher incidence among the elderly, and presents obvious regional and ethnic differences, being more prevalent in populations of East Asian countries. In a systematic review including 129 studies and 1,935 patients, Chen et al. (12) found that 361 cases (18.7%) were diagnosed with TOPLL, 804 cases (41.5%) with TOLF, 143 cases (7.4%) with combined TOPLL and TOLF, and 627 cases (32.4%) with TDH.

Most relevant studies were conducted in China, Japan and the United States. The study also confirmed that TOPLL mainly occurred in the middle thoracic spine (43.4%), TOLF was predominant in the lower thoracic spine (63. 1%), and TDH was mostly located in the middle thoracic spine (46.0%) and lower thoracic spine (50.3%). The predilection for the middle and lower thoracic spine may be attributed to the greater mechanical stress and mobility of these segments. A cross-sectional study conducted by Liang et al. (13) involving 2,000 Chinese subjects showed that the prevalence of TOLF reached 37.65%, while that of thoracic OPLL was 2.25%. In contrast, the prevalence is relatively lower in European and American populations.

Nevertheless, with the widespread use of CT screening, recent American studies have demonstrated that the CT-based prevalence of OPLL ranges from 1.3% to 3.2%, which is lower than 6.3% in Japan and 5.7% in South Korea, indicating that this disease is not exclusive to Asian populations (12, 14, 15).

## 3 Clinical manifestations

TSS has an insidious onset with no obvious symptoms in the early stage, and the condition progresses gradually. Typical clinical manifestations include unilateral or bilateral lower limb pain, numbness and weakness, myelopathic intermittent claudication, band-like constricting sensation in the chest and abdomen, as well as radiating pain distributed along intercostal nerves in the thoracodorsal region and abdominal wall. Some severe patients suffer from defecation and urination dysfunction or sexual dysfunction, which severely reduces the quality of life (16). Physical examination mainly reveals upper motor neuron lesions, manifested as increased muscle tension of unilateral or bilateral lower limbs, active or even hyperactive knee and ankle reflexes, and positive pathological reflexes. When lesions occur in the thoracolumbar segment and cause spinal canal stenosis, mixed lesions of upper and lower motor neurons can be observed, such as enhanced knee reflexes accompanied by weakened ankle reflexes. The core clinical manifestations of TSS are compression of the spinal cord and nerve roots. Clinically, comprehensive judgment combining symptoms, physical signs and imaging examinations is required for early identification and intervention to avoid irreversible impairment of spinal cord function (17).

## 4 Pathological mechanisms

The etiology of TSS is complex and affected by multiple factors including genetics, biomechanics, metabolism and environment. This article mainly discusses the pathological mechanisms ofTOLF, TOPLL and TDH.

### 4.1 Pathological mechanism of thoracic ossification of ligamentum flavum

As the most common pathological basis of TSS, the occurrence and development of TOLF involve the interaction of multiple factors, which ultimately induce the differentiation of flavum ligament fibroblasts into osteogenic cells and lead to heterotopic ossification. Mechanical stress is recognized as a vital triggering factor. The lower thoracic spine, especially the T9-T 12 segments, features high mobility and concentrated stress. Long-term mechanical tensile stress can activate osteogenic signaling pathways such as BMP-Smad1/5/8, Src-p38 MAPK and Wnt/*β*-catenin in the flavum ligament, upregulate the expression of osteogenesis-related genes including Runx2 and Osterix, and further induce the transdifferentiation of flavum ligament cells into osteogenic lineage cells (18, 19). On the other hand, mechanical stimulation can promote local microangiogenesis by regulating the expression of vascular endothelial growth factor (VEGF), increase local microvascular proliferation, and provide essential nutritional and metabolic conditions for ossification (18). Chronic non-specific inflammation also plays a key role in disease progression, serving as an important driving factor for flavum ligament fibrosis, calcification and eventual ossification. Infiltration of inflammatory cells and accumulation of various pro-inflammatory factors can be observed in lesioned tissues of TOLF patients. The research by Huang et al. (20) verified that the level of interleukin-6 (IL-6) was significantly elevated in ossified flavum ligament tissues and the supernatant of primary cultured cells. IL-6 not only functions through the classic JAK/STAT pathway, but also activates the p38 MAPK signaling axis to upregulate the expression of bone morphogenetic protein 2 (BMP2) and osteocalcin, increase the proportion of cells in the S phase of cell cycle, and accelerate the ossification process. In addition, abnormal metabolism of epidural fat is also involved in this process. Exosomal proteomic analysis revealed high expression of lactate dehydrogenase A (LDHA), which leads to lactic acid accumulation and promotes osteogenesis of flavum ligament cells via exosome pathway (21). In conclusion, the local inflammatory microenvironment not only causes tissue damage, but also constructs a pathological microenvironment conducive to the formation of heterotopic ossification. The pathological mechanism of TOLF is a complex process initiated by mechanical force, participated by inflammatory and metabolic factors. Clinically, the concentration of mechanical stress at the highly mobile lower thoracic segments (T9-T 12) explains why TOLF is highly prevalent in these specific regions. The upregulation of inflammatory factors like IL-6 not only accelerates ossification but is also associated with active, progressive-type lesions observed in acute-onset patients. Understanding these mechanical and inflammatory processes helps clinicians identify “active” fast-progressing ossifications on imaging, which may require earlier surgical decompression before irreversible spinal cord ischemia occurs (22, 23). In summary, the pathological mechanism of TOLF is a complex process initiated by mechanical force, participated by inflammatory and metabolic factors, and finely regulated at the epigenetic level.

### 4.2 Pathological mechanism of thoracic ossification of posterior longitudinal ligament

TOPLL is one of the important pathological types leading to thoracic spinal stenosis, which is essentially the ossification of posterior longitudinal ligament fibrous tissues induced by various predisposing factors. Its pathogenesis is complicated, involving the interaction of genetic susceptibility, cellular molecular signal regulation, biomechanics and systemic metabolism. Genetic susceptibility is the core pathogenic factor of TOPLL. Relevant studies have confirmed that TOPLL presents obvious familial aggregation and ethnic differences, with heritability exceeding 53% (24). Genome-wide association studies (GWAS) have identified multiple key susceptible loci. For instance, downregulated expression of R-spondin 2 weakens the inhibitory effect on the Wnt signaling pathway and induces osteogenic differentiation of ligament mesenchymal stem cells (24, 25). Furthermore, Wang et al. (26) confirmed that mutations in the interleukin-17 receptor C gene are closely associated with TOPLL. Such mutations upregulate the expression of interleukin-17 receptor and accelerate the heterotopic ossification of ligament cells. Cellular molecular signaling pathways act as critical mediators in the ossification process. Consistent with ligamentum flavum ossification, the BMP-Smad pathway dominates the initiation of osteogenic differentiation in OPLL. Previous studies have verified that BMP and its receptors are highly expressed in ossified posterior longitudinal ligament tissues, which activate downstream Smad proteins to induce the expression of Runx2, the core osteogenic transcription factor, and further initiate the synthesis of type I collagen and osteocalcin. Meanwhile, abnormal activation of the Wnt/*β*-catenin pathway further promotes the phenotypic transformation of ligament cells from fibroblasts to osteoblasts (27, 28). Located on the posterior edge of vertebral bodies, the thoracic posterior longitudinal ligament bears complex tensile and shear stress due to stress concentration caused by physiological thoracic kyphosis. Long-term chronic mechanical stress stimulates ligament cells to secrete TGF-*β* and BMP-2, activates mechanical stress-sensitive osteogenic signaling pathways, and accelerates calcium salt deposition (27, 29). Another multivariate linear regression analysis confirmed a definite causal relationship between obesity and TOPLL. Besides, patients with TOPLL show systemic hyperosteogeny with significantly higher systemic bone mineral density than age-matched controls, suggesting that TOPLL may be a local manifestation of systemic hyperactive osteogenesis in spinal ligaments (30). To sum up, the pathological process of thoracic posterior longitudinal ligament ossification is a complex process affected by multiple factors and mediated by various mechanisms.

### 4.3 Pathological mechanism of thoracic disc herniation

TDH is mainly caused by annulus fibrosus rupture due to degeneration or external force, resulting in disc herniation into the spinal canal and compression on the thoracic spinal cord or nerve roots. Different from TOLF and TOPLL, the occurrence of TDH is predominantly driven by intervertebral disc degeneration and mechanical factors. TDH accounts for only 0.25%∼ 1% of all intervertebral disc herniations. Given the narrow space of the thoracic spinal canal, herniated lesions are prone to compress the spinal cord and cause severe neurological dysfunction, thus possessing great clinical significance (31). The etiology ofTDH includes degenerative changes, trauma and biomechanical stress. The intervertebral disc consists of the outer annulus fibrosus and inner nucleus pulposus. The annulus fibrosus provides structural support, while the nucleus pulposus rich in proteoglycans and water undertakes the function of pressure buffering. With aging, water loss and proteoglycan degradation occur in the nucleus pulposus, and the collagen fibers of the annulus fibrosus are arranged disorderly, leading to uneven intradiscal pressure distribution. Under chronic cumulative strain or external force, the nucleus pulposus breaks through the annulus fibrosus and protrudes into the spinal canal (32). Mechanical stress is a key predisposing factor. The lower thoracic spine (T8∼T 12) has relatively high mobility and bears large axial load and complex biomechanical force. Long-term micro-injuries accumulate microcracks in the annulus fibrosus, which eventually rupture and lead to nucleus pulposus herniation (33). At the cellular and molecular level, inflammation and matrix degradation are involved in the pathological process of TDH. In degenerative intervertebral discs, annulus fibrosus and nucleus pulposus cells secrete pro-inflammatory factors such as tumor necrosis factor-α (TNF-α) and interleukin-1β (IL-1β). These factors activate matrix metalloproteinases (MMPs) to degrade collagen and proteoglycans and weaken the annulus fibrosus. Meanwhile, local hypoxia and acidic microenvironment further promote cell apoptosis and fibrosis (34). In short, TDH originates from intervertebral disc degeneration, which is aggravated by mechanical stress and inflammation, and eventually progresses to intervertebral disc herniation.

## 5 Pathological classification and imaging evaluation

The pathological classification of TSS is formulated based on the morphological characteristics, distribution range, relationship with the dura mater and spinal cord, as well as lesion size of TOLF, TOPLL and TDH. Pathological classification can not only accurately reflect the disease progression, but also guide the selection of surgical approaches, formulation of decompression strategies and prognosis evaluation.

### 5.1 Pathological classification

#### 5.1.1 Classification of thoracic ossification of ligamentum flavum

TOLF serves as the primary pathological basis ofTSS with widely applied classification systems in clinical practice, among which the Sato classification is the most commonly used (Figure 1). According to the growth direction and progression degree of ossified lesions on CT images, TOLF is divided into five types: lateral type (ossification is limited to the joint capsule and located at the lateral edge of the spinal canal), extended type (ossification is distributed on the surface of flavum ligament and involves interlaminar region without obvious invasion into the spinal canal), enlarged type (ossified masses protrude into the spinal canal without bilateral fusion), fused type (bilateral ossified masses fuse in the midline with a concave section in the center), and nodular type (bilateral ossified masses are completely fused to form nodules protruding into the spinal canal) (35). This classification directly reflects the gradual invasion process of ossification from the lateral side to the midline, and is of great value for evaluating the risk of dural adhesion and selecting surgical methods. Based on sagittal MRI manifestations, Kuh et al. (36) divided TOLF into two types: beak type (ossified masses protrude into the spinal canal in a beak shape with obvious local spinal cord compression) and arc type (ossified masses present round or oval protuberance with mild local spinal cord compression) (Figure 2). The beak type often causes more severe spinal cord lesions and poorer prognosis due to higher local pressure. In addition, Ning et al. (37) classified TOLF into localized type (ossification involving no more than 2 adjacent segments), continuous type (continuous ossification involving 3 or more segments) and intermittent type (discontinuous distribution of localized or continuous ossification) according to MRI features. This classification helps assess the overall severity of multi-segment lesions and determine the range of surgical decompression segments to avoid missed jumping lesions.

**Figure 1:**
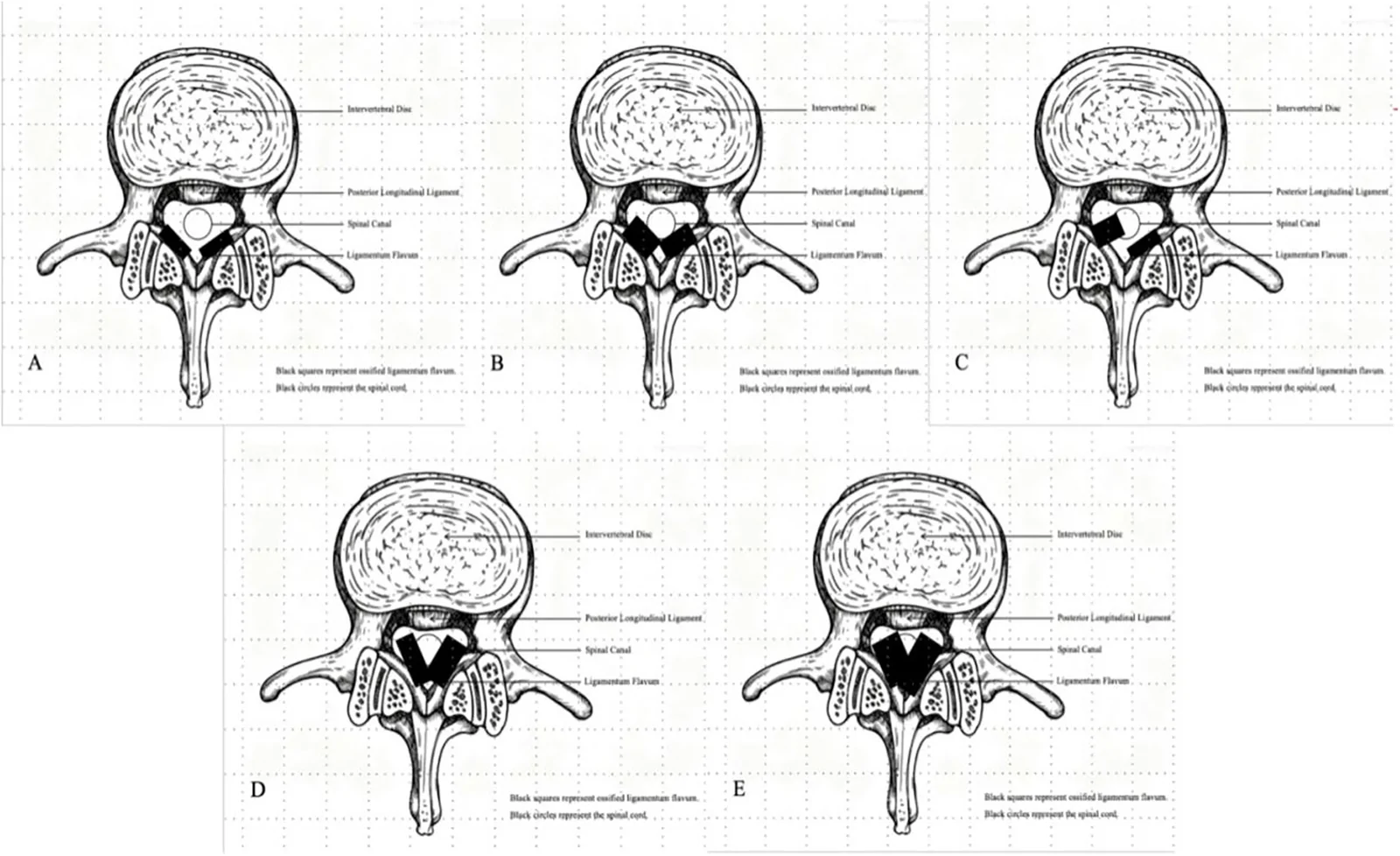
Schematic illustration of sato classification for thoracic ossification of ligamentum flavum (TOLF). **(A)** Lateral type: ossification limited to the joint capsule; **(B)** Extended type: involving the interlaminar region; **(C)** Enlarged type: unilateral protrusion into the canal; **(D)** Fused type: bilateral ossifications fused in the midline with a central notch; **(E)** Nodular type: completely fused nodular mass occupying the canal.

**Figure 2:**
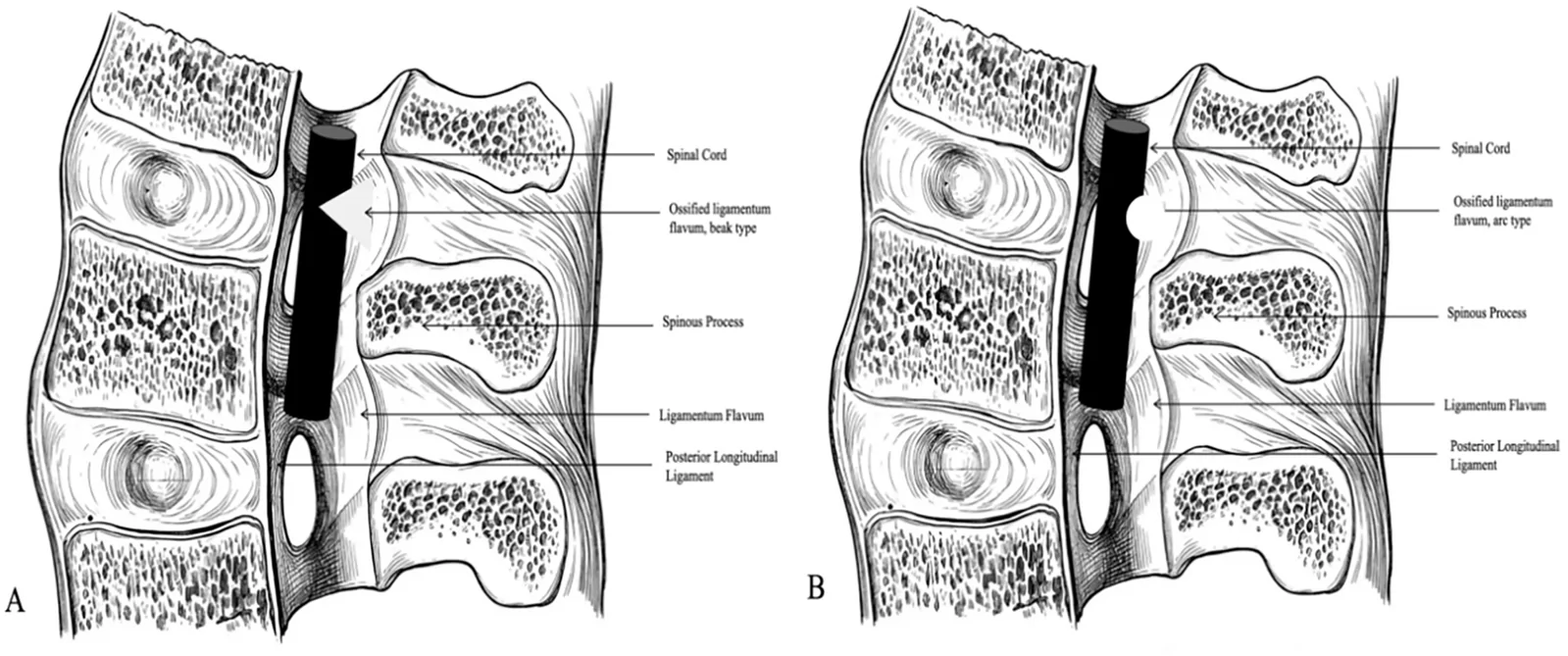
Schematic sagittal illustrations of the kuh classification for thoracic ossification of the ligamentum flavum (TOLF). **(A)** Beak type: Sharp beak-like ossified lesions protrude into the spinal canal and cause severe focal compression of the thoracic spinal cord, which is associated with serious myelopathic lesions and unfavorable prognosis. **(B)** Arc type: Smooth round/arc-shaped ossified bulges exert mild compression on the spinal cord with relatively slight neurological dysfunction.

#### 5.1.2 Classification of thoracic ossification of posterior longitudinal ligament

The classification of TOPLL mostly follows the classic classification system of cervical ossification of posterior longitudinal ligament. According to sagittal x-ray or CT images, the Ministry of Health, Labour and Welfare of Japan classified TOPLL into continuous type (uninterrupted lesions involving multiple segments), segmental type (multiple independent lesions at vertebral levels), localized type (isolated lesions in single vertebral segment) and mixed type (combination of the above three types) (38, 39). Affected by physiological thoracic kyphosis, TOPLL is mostly manifested as segmental type or mixed type. Abiola et al. (25) further subdivided TOPLL into flat type and rostral protrusion type. The latter is more likely to cause irreversible spinal cord injury due to local stress concentration. In recent years, by analyzing clinical outcomes and surgical complications of 108 TOPLL patients, Liu et al. (40) proposed a surgical decision-making classification algorithm for TOPLL, which comprehensively considers ossification type, segmental location and spinal cord displacement. This algorithm significantly improves the accuracy of surgical approach selection for decompression surgery of thoracic posterior longitudinal ligament ossification.

#### 5.1.3 Classification of thoracic disc herniation

Traditional classification of TDH is based on lesion location, including central type, paracentral type and lateral type; it can also be divided into soft herniation and calcified herniation according to lesion properties. Calcified herniation is more common in the upper and middle thoracic spine, which is prone to adhere to the dura mater and brings great surgical difficulty. In recent years, Farber et al. (41) established a novel classification system: type 0 refers to small disc herniation (occupying ≤40% of the spinal canal) without obvious spinal cord or nerve root compression; type 1 represents small paracentral disc herniation; type 2 is small central disc herniation; type 3 indicates huge paracentral disc herniation (occupying >40% of the spinal canal) that severely displaces the spinal cord to the contralateral side with prominent clinical symptoms; type 4 is huge central disc herniation causing severe spinal cord compression. Subtypes can be further divided according to calcification status, which is conducive to predicting the degree of intraoperative neurological damage and guiding surgical approach selection. It is worth noting that TOLF, TOPLL and TDH often coexist clinically to form complex mixed-type TSS. Under such circumstances, comprehensive evaluation of segmental distribution, compression orientation, calcification degree and spinal cord compression status is required to identify responsible compression segments and main pathological mechanisms, so as to formulate individualized surgical strategies.

### 5.2 Imaging evaluation

In orthopedics, x-ray radiograph remains an essential basic examination for preliminary screening of TSS. It can detect signs of ossification on the posterior vertebral edge or interlaminar region and changes of intervertebral space, assist in judging combined anatomical abnormalities, provide preoperative reference information for surgeons and facilitate intraoperative lesion localization (42, 43).

Nevertheless, x-ray has low sensitivity and specificity and cannot make a definite diagnosis. At present, CT and MRI are the main diagnostic methods for TSS in clinical practice. With advanced image post-processing technology, CT can clearly display the specific location and morphology ofTOLF and TOPLL, the degree of thoracic spinal canal stenosis and the scope of dural ossification (44). On axial CT images, TOLF is mostly presented as high-density ossified shadows distributed along vertebral laminae or facet joint capsules, and bridge-like ossification is commonly seen on sagittal CT images; while TOPLL manifests as linear or crescent-shaped osseous high-density shadows on the posterior side of vertebral bodies on sagittal CT images44. Benefiting from excellent soft tissue imaging performance, MRI can clearly show flavum ligament hypertrophy, disc herniation and spinal cord compression.

Clinically, combined application ofCT and MRI is commonly adopted: CT clarifies osseous anatomical details, and MRI evaluates the degree of spinal cord and nerve damage. The combined application can increase the diagnostic accuracy to more than 95% (45, 46). In conclusion, preoperative pathological classification and imaging evaluation are the keys to precise diagnosis and treatment of TSS, which provide core basis for surgical approach selection and decompression scheme formulation, and reduce the risk of complications such as dural rupture and nerve injury, especially for the diagnosis and treatment of complex mixed-type TSS.

## 6 Treatment

The treatment of TSS should follow the individualized principle based on comprehensive evaluation of symptom severity, pathological basis and spinal cord compression status. This article summarizes traditional open surgery and minimally invasive spinal techniques for TSS treatment to provide references for clinical practice (Table 1).

**Table 1:** Comparison of surgical outcomes and complications for thoracic spinal stenosis treatments.

Surgical approach	Main clinical indications	Expectable Outcomes (JOA recovery rate)	Com plication rates (key types)	Reoperation rate	Key advantages	Key disadvantages
Posterior Laminectomy(with/without fusion)	•Multi-segmentTOLF/TOPLL •Severe canal stenosis with duralossification	50%–70% (Significant long-term neurological relief)	10%–25% •CSFL:11%–15% •PND: 5%–10% •Wound infection	2%–5%	•Thorou gh decompression under direct vision • Familiar technique with wide surgical field	• High trauma to posterior musculature •Risk of postoperative kyphosis if unfused
Flip-type posterior resection(En Bloc/Bridge Crane)	•Massive, fused,or nodular TOLF • High risk of dural adhesion	55%–75% (Excellent decompression of posterior wall)	15% – 22% •CSFL: ∼22% •Transient neurological deficit	1%–3%	• Avoids directly instrum enting highly compressed areas • Relieves circular adhesion safely	• High technical difficulty • High rate of CSFL due to enblocdural peeling
Hemilamin ectomy/ULBD	• Unilater al or localized TOLF • Single-segmentstenosis	70%–83% (Excellent localize d recovery)	3% – 8% • Local Hematoma • Low CSFL risk (<5%)	<2%	• Minimizes damage To posterior column stability •Signific ant reducti on in blood loss	• Narrower visual field • Steep learning curve for bilateral decompression
Anterior Decompression (Trans-sternal/Extrapleural)	• Severe anterior compression (TOPL L, TDH) •Rigid, localized ventralmasses	30%–55% (Highly variable depending on dural adhesion)	30%–45% •CSFL:∼31.6% • PND:∼ 10.5% • Pleuraleffusion	5%–8%	•Anatomically direct removal of ventral compression	•Extreme surgical trauma • High risk of catastrophic spinal cord injury
Posterior Indirect Decompression (with instrumented fusion)	• Multi-segment TOPLL • Kyphoticthoracicdeformity	50%–65%(Gradual spinal cord drift-bac k)	5%–12% •Screw malposition • Moderate CSFL (<10%)	2%–4%	• Avoids separating dense ventral dural adhesions • Restores sagittal alignment	• Does not remove the ossified mass directly • Limited efficacy in rigid, flat-type OPLL
Minimally Invasive Endoscopy(PE D/UBE/AUSS/OSE)	• Single-segment TOLF/ TDH • Elderly patients with multiple comorbidities	75%–85% (Rapid symptomatic improvement)	2%–5% • Rare CSFL (<3%) •Transient nerve root irritation	<2%	• Minimal tissue trauma and blood loss • Can be performed under local anesthesia	• Very steep Learning curve • High risk of incomplete decompression in fused types

### 6.1 Conservative treatment

For early-stage TSS patients with mild symptoms, non-surgical conservative treatment can be preferentially adopted, including bed rest, physical rehabilitation therapy, non-steroidal anti-inflammatory drugs and neurotrophic agents. Since the pathological basis of most TSS cases is rigid compressors such as TOLF and TOPLL, conservative treatment can only temporarily relieve symptoms rather than eliminate intraspinal compression, failing to achieve long-term therapeutic effects. Therefore, conservative treatment is mainly applied for early observation or perioperative adjuvant therapy. Surgical intervention should be performed once symptoms progress gradually to avoid missing the optimal treatment opportunity (46).

### 6.2 Thoracic spinal stenosis caused by posterior spinal cord compression

Traditional open surgery is a classic treatment for TSS, which is especially suitable for patients with multi-segment lesions, kyphotic deformity and dural ossification. Its core objectives are to achieve sufficient spinal cord decompression and maintain or reconstruct spinal stability. Classic surgical procedures include posterior median total laminectomy, flip-type posterior wall resection, hemilaminectomy and laminotomy, which are often combined with pedicle screw internal fixation to maintain spinal stability. Total laminectomy is widely recognized as the current gold standard for TSS treatment. Via posterior median approach, lesions are exposed, and laminectomy extending 1–2 segments above and below the lesions is performed, ossified flavum ligament lesions are removed, and partial bilateral facet joints (about 1/3∼ 1/2) are resected to achieve direct decompression. Wang et al. (45) performed total laminectomy on 142 patients, who were divided into simple laminectomy group and laminectomy combined with internal fixation group. After 2 years of follow-up, no neurological deterioration occurred in all patients, and the Japanese Orthopaedic Association (JOA) scores of both groups were significantly improved compared with preoperative levels. However, dural tear occurred in 16 patients in total during the operation, mainly due to severe adhesion between ossified masses and dura mater that cannot be separated and resected independently. Zhong et al. (47) conducted a retrospective analysis on 22 patients undergoing laminectomy. The final follow-up results showed that the average JOA score increased significantly from 5.64 preoperatively to 9.18, and the Visual Analogue Scale (VAS) score decreased from 3.08 to 1.05, achieving satisfactory therapeutic effects, whereas surgical-related complications occurred in 9 cases with a relatively high incidence rate. The flip-type thoracic spinal canal posterior wall resection is a commonly used modified technique in China. During the operation, high-speed burr or ultrasonic bone scalpel is used to make grooves along the medial side of bilateral articular processes to disconnect the connection between ossified posterior and lateral walls of the spinal canal. Afterwards, the posterior wall is slowly lifted from one end to separate adhesions with the dural sac, and finally *en bloc* resected just like opening a cover. Sun et al. (48) performed flip-type posterior wall resection on 44 TSS patients. At the final follow-up, the JOA score was significantly elevated from 6.68 ± 1.76 to 8.84 ± 1.83. Postoperative MRI and CT confirmed sufficient decompression range, and dural tear occurred in 10 cases, all of which were relieved after conservative treatment. With the development of medical technology, three-dimensional digital technology has been applied in clinical practice for preoperative reconstruction of ossified lesions and simulated segmented resection, which has achieved favorable clinical efficacy and improved the precision, safety and effectiveness of open surgery for TOLF (49). For cases dominated by unilateral compression, hemilaminectomy can significantly reduce the destruction of spinal stability and lower the incidence of surgical complications. Via posterior median incision, only the affected side is exposed to resect the affected lamina, ossified masses and medial 1/3 of facet joints, while reserving the contralateral lamina and facet joints to realize unilateral laminotomy for bilateral decompression (ULBD). Kim et al. (50) retrospectively analyzed 11 TOLF patients treated with hemilaminectomy and bilateral decompression. Postoperatively, the JOA scores were obviously improved, and the lower limb muscle strength and sensory function recovered well in most patients. The cross-sectional area of the dural sac increased from an average of 57.56 mm^2^ preoperatively to 111.8 mm^2^ postoperatively, with a significantly lower complication rate than total laminectomy. Tumturk et al. (51). adopted the same surgical protocol to treat 21 TSS patients with an average follow-up of 37.6 months. The average JOA score increased from 5 preoperatively to 10 at the final follow-up with a recovery rate of 83.3%, and no postoperative spinal instability was observed during follow-up, confirming reliable therapeutic effects. Laminotomy is more suitable for single-segment localized lesions with minimal trauma. High-speed burr is used to make fenestration between lesioned laminae, thin the lamina and resect ossified lesions with rongeur to achieve precise decompression without extensive laminectomy. Eun et al. (52) applied laminotomy decompression in 17 TOLF patients. The average preoperative JOA score was 4.88, which rose to 7.00 postoperatively with remarkable symptom improvement, and only 1 case of dural tear was observed, presenting high overall safety. In summary, preoperative accurate pathological classification is essential before open surgery. Total laminectomy and flip-type resection are preferred for enlarged type, nodular type, fused type and multi-segment lesions to ensure thorough decompression; hemilaminectomy or laminotomy can be adopted for lateral type, extended type and single-segment localized lesions to reduce posterior column destruction, lower complication incidence and decrease the risk of spinal instability. Clinical treatment should be individualized to select the optimal surgical scheme according to specific patient conditions.

### 6.3 Thoracic spinal stenosis

Caused by Anterior Spinal Cord Compression Anterior spinal cord compression is mainly induced by TOPLL and TDH, among which TOPLL is more common and mostly involves the upper and middle thoracic spine.

According to lesion location, involved segment number and spinal cord displacement degree, optional surgical methods include anterior TOPLL resection, posterior indirect decompression and circumferential decompression. TDH-induced thoracic spinal stenosis has a low incidence and is mostly treated with minimally invasive spinal surgery, which will not be elaborated in this section. Anterior approach for ossified posterior longitudinal ligament resection is anatomically the most reasonable method to completely relieve ventral spinal cord compression and achieve direct decompression, including trans-sternotomy approach and extrapleural approach.

However, this approach is characterized by complex anatomical exposure, severe trauma and high technical requirements for surgeons. Min et al. (53) performed anterior decompression surgery on 19 TOPLL patients. The average JOA score increased from 4.9 preoperatively to 6.3 at the final follow-up with a JOA improvement rate of 34.2%, while the complication rate reached 42. 1%, mainly including cerebrospinal fluid leakage (31.6%) and neurological deterioration (10.5%). Hanai etal (54). adopted direct anterior resection of ossified posterior longitudinal ligament in 12 TOPLL patients, and the average JOA score increased from 5.3 ± 0.4 preoperatively to 6.9 ± 0.5 at the final follow-up, verifying favorable therapeutic effects of anterior TOPLL resection. Nevertheless, anterior approach requires sternotomy or rib resection with narrow surgical visual field, which is easy to cause damage to thoracic and abdominal visceral organs, and may aggravate spinal cord injury during decompression for severe ventral spinal cord compression, thus it is rarely applied in current clinical practice. Posterior indirect decompression has become the mainstream surgical method for anterior compression, which is technically less difficult and suitable for multi-segment lesions. Koda etal (55). performed posterior indirect decompression and fusion surgery on 20 TOPLL patients with 5 years of follow-up. The average JOA score increased by 3.8 points, and only 2 cases of complications occurred. The research team confirmed that posterior indirect decompression and fusion is a relatively safe and stable surgical method for TOPLL treatment. Surgical timing is crucial for the prognosis of TOPLL patients. A prospective single-center study involving 71 cases indicated that early implementation of posterior indirect decompression, sufficient intraoperative spinal cord decompression combined with intraoperative neurophysiological monitoring is conducive to improving surgical outcomes (56). Relevant reviews also demonstrated that posterior approach features clear surgical field and large operating space, which can realize indirect anterior decompression via laminectomy and laminoplasty with less trauma and fewer complications compared with anterior approach (57). It should be noted that simple posterior decompression for rostral protrusion-type TOPLL may aggravate kyphosis and further deteriorate spinal cord injury, so pedicle screw fixation system is recommended in clinical practice (58). Posterior indirect decompression avoids stripping dense adhesions between ossified ligaments and dural sac, thus reducing the risk of intraoperative spinal cord injury, and is especially applicable to patients with high surgical risks of anterior resection. Circumferential decompression is indicated for massive TOPLL or mixed lesions combined with TOLF to relieve both anterior and posterior compression simultaneously. Circumferential decompression can be completed via simple posterior approach: extended laminectomy is performed firstly, then ossified posterior longitudinal ligament is removed or floated via posterolateral V-shaped excavation to achieve 360° decompression, followed by intertransverse process bone graft fusion and internal fixation (57, 59). After establishing sufficient ventral decompression space, ossified posterior longitudinal ligament is resected and taken out through the preoperatively constructed tunnel. A total of 26 TOPLL patients were enrolled in this study, and no complications were observed during 1-year postoperative follow-up, which verified the safety and efficacy of this surgical technique (60). In conclusion, although anterior direct decompression achieves thorough decompression anatomically, its clinical application has been greatly limited due to complex surgical approach, narrow visual field and high complication rate. Posterior indirect decompression combined with internal fixation and circumferential decompression have become mainstream surgical methods at present. Regardless of the selected surgical approach, intraoperative neurophysiological monitoring is recommended to dynamically monitor intraoperative spinal cord and nerve function and reduce the incidence of postoperative neurological deterioration.

### 6.4 Minimally invasive spinal techniques

With the development of surgical instruments and in-depth promotion of enhanced recovery after surgery concept, the treatment of TSS is transforming from conventional open surgery to minimally invasive surgery with minimal incision. On the premise of ensuring adequate decompression, these techniques maximize soft tissue protection and preserve spinal stability. According to surgical instrument types, minimally invasive techniques are roughly divided into single-channel endoscopy and dual-channel endoscopy systems. Percutaneous endoscopic decompression (PED) is the most widely applied technique in single-channel endoscopy system. Initially used for the treatment of lumbar disc herniation, it has been gradually applied in the treatment of full-spine diseases.

Ruetten etal (61). firstly reported the application of single-channel endoscopy in 55 TSS patients, and confirmed that endoscopic surgery can preserve the integrity of vertebral laminae and epidural fat without extensive tissue destruction, thus reducing the risk of dural adhesion. Cheng et al. (62) performed percutaneous endoscopic thoracic decompression under local anesthesia in 12 TSS patients including 5 cases ofTOLF, 5 cases of TOPLL and 2 cases ofTDH. Only 1 case of dural tear occurred during the operation, and the average JOA score increased significantly from 6.25 ± 1.60 preoperatively to 9.75 ± 1.21 at the final follow-up. This technique has the advantages of minimal trauma, rapid recovery and low postoperative infection rate, and can be performed under local anesthesia, which is especially suitable for elderly patients with poor cardiopulmonary function, multiple underlying diseases and slow physical recovery. With the popularization of spinal minimally invasive surgery, an increasing number of domestic clinical studies have reported the application of PED in TSS treatment. Huang etal (63). applied interlaminar PED in 24 TOLF patients. The average preoperative VAS score was 6.2 ± 0.9, which decreased significantly to 1.5 ± 0.5 at the final follow-up with statistically significant difference. According to the modified Macnab efficacy evaluation criteria, the excellent and good rate reached 83.3%, and all patients recovered well without surgical-related complications. Numerous previous studies have confirmed that PED treatment for TSS is characterized by minimal trauma, short hospital stay and favorable clinical efficacy with significant improvement of clinical scale scores (64, 65). When performed under local anesthesia, patients can timely feedback intraoperative discomfort to surgeons to reduce complication risks. However, this technique is limited by narrow operating space and strict anatomical requirements with a steep learning curve, and inexperienced surgeons may fail to achieve sufficient cephalic and caudal decompression range. Unilateral biportal endoscopy (UBE) is the most representative technique in dual-channel endoscopy system, which provides larger operating space and allows efficient and safe removal of ossified masses using high-speed burr. Yang etal (66). treated 11 TOLF patients with UBE, and all patients achieved relieved symptoms without surgical complications. Postoperative CT measurement showed that the average anteroposterior diameter of the spinal canal increased from 3.67 ± 1.37 mm preoperatively to 8.63 ± 1.61 mm postoperatively with statistical significance, and both JOA and VAS scores were obviously improved after surgery. Wang etal (67). conducted a retrospective study on TOLF patients divided into UBE group and open surgery group. Both groups achieved satisfactory postoperative therapeutic effects, while UBE was superior to open surgery in terms of hospital stay, intraoperative fluoroscopy times, incision length and off-bed activity time, confirming that UBE can achieve equivalent therapeutic effects as open surgery with less trauma and faster recovery. In recent years, a series of independently developed domestic dual-channel endoscopic techniques have emerged, such as arthroscopic-assisted uni-portal spinal surgery (AUSS) and one-hole split endoscopy (OSE). Essentially consistent in technical principle, they differ from UBE in that the observation channel and operating channel are set in the same incision rather than two separate incisions, which is more in line with the operational habits of open surgery and easier to master for spinal surgeons lacking minimally invasive surgical experience. Sha et al. adopted OSE technique in 13 TOLF patients. Postoperative imaging confirmed complete removal of ossified lesions, and the lower limb VAS score decreased from 6.5 ± 0.8 preoperatively to 1.6 ± 0.4 at the final follow-up. No complications such as cerebrospinal fluid leakage and spinal cord injury occurred, preliminarily verifying the clinical promotion value of this novel technique, while long-term follow-up is still needed to observe its long-term therapeutic efficacy (68). In summary, minimally invasive spinal techniques have shown prominent advantages of minimal trauma, rapid recovery and low complication rate in TSS treatment, serving as powerful supplements to open surgery and becoming mainstream clinical surgical methods.

## 7 Summary and prospect

TSS is less common than lumbar spinal stenosis with overlapping partial clinical symptoms, which easily leads to missed diagnosis in clinical practice. Conservative treatment is mostly ineffective once patients develop myelopathic symptoms, and timely surgical intervention is required. At present, various surgical schemes, approaches and novel technologies have been reported in relevant literatures, yet there is no unified conclusion on the optimal surgical method. Individualized treatment strategies should be formulated based on spinal cord compression status and pathological classification. Open surgery allows lesion resection and decompression under direct vision with thorough decompression effect, but it inevitably destroys spinal stability and causes severe trauma due to wide surgical exposure. In recent years, percutaneous endoscopic surgery has developed rapidly in China with a large number of clinical cases and published researches ranking among the world's leading level, and has been widely applied in the treatment of various spinal diseases. Compared with open surgery, percutaneous endoscopy has obvious advantages in terms of surgical trauma, intraoperative fluoroscopy times and complication rate, while its shortcomings cannot be ignored, including steep learning curve, limited intraoperative operating space and visual field, as well as limitations in contralateral decompression. The author holds that minimally invasive treatment of TSS should adhere to the principles of safety priority and precise operation, and avoid sacrificing decompression thoroughness for blind pursuit of minimal invasiveness.

Open surgery remains irreplaceable for patients with multi-segment massive ossification, severe kyphosis or obvious spinal instability. With the rapid development of medical-industrial cross-field technology in the AI era, artificial intelligence can realize automatic quantitative analysis ofpreoperative CT/MRI images through deep learning algorithms, accurately delineate ossified boundaries, calculate spinal canal occupancy rate and predict spinal cord floating degree, so as to provide objective data support for individualized treatment formulation. Intraoperative surgical instruments should avoid direct contact with the spinal cord, and surgical robots can eliminate hand tremor of surgeons to realize submillimeter-level precise operation and greatly improve the safety of decompression surgery. With the further development of science and technology as well as surgical instruments, patients with TSS will have more optimized surgical options and better clinical therapeutic effects in the future.
